# Improving prevention, early recognition and management of acute kidney injury after major surgery: results of a planning meeting with multidisciplinary stakeholders

**DOI:** 10.1186/s40697-014-0020-y

**Published:** 2014-08-26

**Authors:** Matthew T James, Elijah Dixon, Derek J Roberts, Rebecca Barry, Carlee Balint, Aleem Bharwani, William Don Buie, Tomas Godinez, Brenda R Hemmelgarn, John B Kortbeek, Braden J Manns, Andria Marin, Nairne Scott-Douglas, Henry Tom Stelfox, Neesh Pannu

**Affiliations:** Department of Medicine, University of Calgary, Calgary, Alberta Canada; Department of Community Health Sciences, University of Calgary, Calgary, Alberta Canada; Department of Surgery, University of Calgary, Edmonton, Alberta Canada; Department of Critical Care, University of Calgary, Calgary, Alberta Canada; Department of Medicine, University of Alberta, Edmonton, Alberta Canada; Division of Nephrology, Departments of Medicine and Community Health Sciences, University of Calgary, Calgary, AB T2N 2T9 Canada

## Abstract

**Purpose of review:**

Acute kidney injury (AKI) is common after major surgery, and is associated with morbidity, mortality, increased length of hospital stay, and high health care costs. Although recent guidelines for AKI provide recommendations for identification of patients at risk, monitoring, diagnosis, and management of AKI, there is lack of understanding to guide successful implementation of these recommendations into clinical practice.

**Sources of information:**

We held a planning meeting with multidisciplinary stakeholders to identify barriers, facilitators, and strategies to implement recommendations for prevention, early identification, and management of AKI after major surgery. Barriers and facilitators to knowledge use for peri-operative AKI prevention and care were discussed.

**Findings:**

Stakeholders identified barriers in knowledge (how to identify high-risk patients, what criteria to use for diagnosis of AKI), attitudes (self-efficacy in preventive care and management of AKI), and behaviors (common use of diuretics, non-steroidal anti-inflammatory drugs, withholding of intravenous fluids, and competing time demands in peri-operative care). Educational, informatics, and organizational interventions were identified by stakeholders as potentially useful elements for future interventions for peri-operative AKI.

**Limitation:**

Meeting participants were from a single centre.

**Implications:**

The information and recommendations obtained from this stakeholder’s meeting will be useful to design interventions to improve prevention and early care for AKI after major surgery.

## Abrégé

**Objectif de l’étude:**

L’insuffisance rénale aiguë (IRA) est fréquente à la suite d’une chirurgie importante et elle est associée à une morbidité, à une mortalité, à une hospitalisation prolongée et à des coûts élevés liés aux soins de santé. Bien que les lignes directrices récentes concernant l’IRA fournissent des recommandations pour déterminer les patients à risque, de même que pour contrôler, diagnostiquer et prendre en charge l’IRA, la compréhension fait défaut pour mener leur mise en place réussie dans la pratique clinique.

**Sources d’information:**

Nous avons tenu une réunion de planification avec des acteurs pluridisciplinaires afin de cibler les obstacles, les appuis et les stratégies de mise en œuvre des recommandations pour la prévention, l’identification précoce et la prise en charge de l’IRA suite à une chirurgie importante. On a abordé les obstacles et les appuis à l’utilisation des connaissances dans la prévention périopératoire de l’IRA et les soins qui s’y rattachent.

**Résultats:**

Les acteurs ont déterminé les obstacles à la connaissance (comment identifier les patients à risque élevé, le choix de critères diagnostiques pour l’IRA), les attitudes (l’auto-efficacité dans les soins préventifs et la prise en charge de l’IRA), et les comportements (l’usage courant de diurétiques, d’anti-inflammatoires non stéroïdiens, la non-administration de solutés intraveineux, et les contraintes de temps dans les soins périopératoires). Les acteurs ont défini les interventions éducatives, informatiques et organisationnelles comme des éléments potentiellement utiles dans les interventions futures en soins périopératoires pour l’IRA.

**Limites de l’étude:**

Les participants à la réunion provenaient d’un seul et même centre.

**Impacts:**

Les informations et recommandations obtenues au cours de la réunion des acteurs seront utiles pour l’élaboration des interventions afin d’améliorer la prévention et les soins précoces relatifs à l’IRA suite à une chirurgie majeure.

## Why is this report/review important?

Acute kidney injury (AKI) is common, and the incidence of AKI treated with dialysis after major surgery has been rising in Canada. Although guidelines have recently been published for AKI, their successful uptake requires engagement with providers from multiple disciplines.

## What are they key messages?

Stakeholders identified several barriers in knowledge, attitudes, and behaviors related to prevention, early identification, and management of AKI after major surgery. Educational, informatics, and organizational interventions were identified as potentially useful strategies for future implementation and evaluation.

## Implications for future research/policy

The information from this report can help tailor the design of future educational, informatics, and organizational interventions to implement guidelines for AKI in postoperative care settings.

## Introduction

Acute kidney injury (AKI) has an incidence of 1% following all types of surgery, but occurs much more frequently after cardiac, vascular, and intra-abdominal surgeries where the incidence ranges from 10-30% [1]. Like AKI in other settings, peri-operative AKI is associated with increased morbidity, mortality, length of hospital stay, and healthcare costs; and dialysis may be required in severe cases [2,3]. The incidence of AKI has risen 4-fold over the past 20 years in North America [4,5], including a greater than 3-fold increase in the incidence of AKI treated with acute dialysis after major surgery in Canada [6].

Risk factors for AKI after major surgery include non-modifiable factors such as age, comorbidities, and surgery type, as well as potentially modifiable risk factors such as intravascular volume depletion, hypoperfusion, pre- and peri-operative medication use, and exposure to intravascular contrast agents [2]. Medications commonly used in the peri-operative period that increase the risk of AKI include non-steroidal anti-inflammatory drugs (NSAIDs), angiotensin converting enzyme (ACE) inhibitors, angiotensin receptor blockers (ARBs), diuretics, and aminoglycoside antibiotics [2,7]. Despite an understanding of the modifiable risk factors that predispose to AKI, translation of this knowledge into improvements in care and outcomes of AKI has been limited.

Recent guidelines for AKI provide diagnostic criteria (Table 1), recommendations for identification of high risk patients, appropriate monitoring, and strategies to ensure timely recognition of AKI [8,9]. However, implementation of these recommendations into clinical practice remains challenging because patients who develop AKI are generally not under the care of kidney specialists. Successful uptake requires engagement with providers from multiple disciplines including nursing, surgery, internal medicine and critical care. Deficiencies in knowledge and clinical practice in prevention, early recognition, and management of AKI are common [10,11].

**Table 1:** **Kidney Disease Improving Global Outcomes (KDIGO) definition of AKI**

**Stage**	**Serum creatinine**	**Urine output**
**1**	1.5–1.9 times baseline or ≥26.5 μmol/L increase	<0.5 ml/kg/h for 6–12 hours
**2**	2.0–2.9 times baseline	<0.5 ml/kg/h for ≥12 hours
**3**	3.0 times baseline or increase to ≥353.6 μmol/L or initiation of renal replacement therapy	<0.3 ml/kg/h for ≥24 hours or anuria for ≥12 hours

*Abbreviation:* *AKI* Acute kidney injury.

A recent review of the Kidney Disease Improving Global Outcomes (KDIGO) AKI guidelines by the Canadian Society of Nephrology (CSN) highlighted the need to develop knowledge translation strategies to increase uptake of recommendations regarding prevention and management of AKI in Canada [12]. AKI after major surgery is a prime candidate condition to target because it is of clinical concern, standards for identification and management of the condition exist, and recent data identify evidence-to-practice gaps [2,13]. In this article we review information from a planning meeting with multidisciplinary stakeholders to identify barriers, facilitators, and strategies to increase uptake of recommendations to improve prevention, early identification, and management of AKI after major surgery.

## Review

### Methods

#### Overview of stakeholder meeting

A planning meeting with 15 participants was held in Calgary, Alberta in October 2013. Recognizing that care of patients with AKI after surgery involves providers from several stakeholder groups, participants included physician and nursing practitioners from medicine, surgery, critical care, and nephrology along with representatives with cross-disciplinary expertise in health administration, health services research, pharmacy, and implementation science/knowledge translation (Table 2). The key objectives of the meeting were to review the evidence-to-practice gaps that exist for AKI after major surgery in Alberta, identify barriers and facilitators to prevention, early recognition, and management of AKI after major surgery, and to identify potential interventions tailored to address these clinical challenges.

**Table 2:** **Demographic and professional characteristics of the meeting participants**

**Characteristic**	**n (%) N = 15**
Age (years)	
30-39	5 (33.3)
40-49	5 (33.3)
≥50	5 (33.3)
Gender	
Female	5 (33.3)
Professional Background^a^	
Physician	11 (73.3)
Researcher	8 (53.3)
Administrator	3 (20.0)
Nurse	1 (6.7)
Pharmacist	1 (6.7)

^a^Participants may be counted more than once.

### Meeting activities

The meeting consisted of four components. Following the introduction of all participating stakeholders and an overview of the objectives of the meeting, a presentation was given summarizing the current state of knowledge about AKI risk factors, diagnostic criteria, management, and outcomes with an emphasis on recent recommendations provided by the KDIGO AKI guidelines, National Institute for Clinical Excellence (NICE) AKI guidance, and the CSN commentary on AKI guidelines [8,9,12]. Next, to highlight local variation in practice and evidence to care gaps, data on the local incidence of AKI and processes of care after major surgery from local hospitals (Calgary, Canada) were reviewed. Further, the results of a pre-meeting survey of 20 local clinicians from surgery, critical care and nephrology were reviewed, to examine local opinions about agreement with peri-operative AKI prevention, monitoring, and management recommendations from the published guidelines [8,9]. The third component of the meeting was a discussion about barriers and facilitators to uptake of recommendations for AKI prevention and management. The final component involved a facilitated group discussion about interventions and potential strategies that might close local evidence-to-practice gaps for AKI prevention and care. Potential interventions were presented according to the Effective Practice and Organization of Care (EPOC) Taxonomy of Interventions [14], developed by the Cochrane Collaboration, and included formal education, linkage and exchange, audit and feedback, informatics, patient-direct/patient-mediated interventions, organizational, shared decision making, and financial incentives [13]. Participants were encouraged to ask questions and initiate discussions throughout the meeting. The meeting was recorded and content was qualitatively summarized by two individuals to identify major themes and recommendations from the discussion. Ethics approval was obtained from University of Calgary Conjoint Health Research Ethics Board. All participants reviewed the final manuscript and agreed upon the content and recommendations.

### Results

#### Knowledge of local context

**Figure 1:**
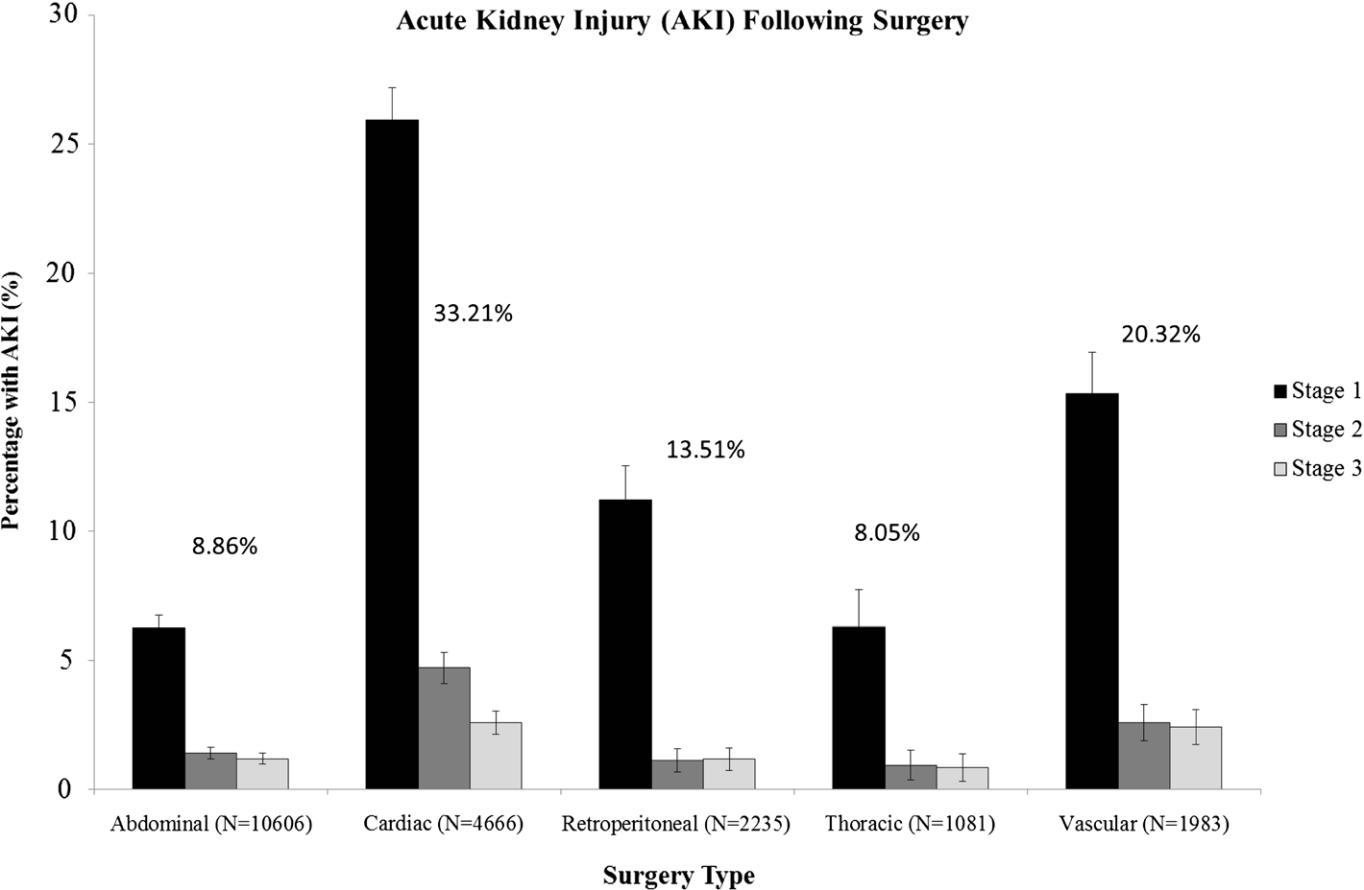
**Incidence of AKI, according to surgery type, in Calgary Alberta, between 2005–2012.** The incidence is shown for each of the stages of AKI based on serum creatinine changes according to the KDIGO AKI definition. The incidence of all stages of AKI is shown above the bars for each type of surgery. Abbreviations: AKI = acute kidney injury.

The incidence of AKI (identified based on serum creatinine changes and defined according to the KDIGO definition [9]) after major surgery between 2005 and 2012 in Calgary Zone hospitals ranged from 8.0% after thoracic surgery to 33.2% after cardiac surgery (Figure 1). Urine output was recorded in the electronic medical record on each shift for 15-25% of patients on surgical wards on each of the 7 days following surgery, and 45-60% of patients had fluid balance recorded each day on each of the first 7 post-operative days. Medications and intravenous fluids prescribed within 24 hours of the onset of AKI were also examined, according to the type of surgery performed (Table 3). Diuretics were prescribed within 24 hours of AKI onset to between 6 to 38% of patients, while 20-61% of patients were administered intravenous crystalloids within 24 hours of AKI recognition. Between 3 to 14% of patients with AKI were prescribed an NSAID, and 3 to 18% of patients were prescribed an ACE inhibitor or ARB within 24 hours after AKI onset.

**Table 3:** **Opinions of care providers about medications use after onset of AKI and the proportions of these medications prescribed within 24 hours of AKI, according to type of major surgery in Calgary, Alberta, between 2005-2012**

**Survey**		**% of Patients that were Prescribed Medication within one day of AKI onset, by surgery type**					
**Questions**	**Physicians that agreed, n (%)**		**Abdominal, %, (N = 941)**	**Cardiac, %, (N = 1550)**	**Retroperitoneal, %, (N = 302)**	**Thoracic, %, (N = 87)**	**Vascular, %, (N = 403)**
Diuretics should be avoided in the absence of volume overload after the onset of AKI	19 (95)	Diuretics	15	38	6	16	25
Patients with AKI should not receive NSAIDs after the onset of AKI	19 (95)	NSAID	6	7	8	14	3
Patients with AKI should avoid ACEI and/or ARB drugs after the onset of AKI	12 (60)	ACEI or ARB	5	18	3	6	18
Intravenous crystalloids should be used to optimize hemodynamic status and restore effective circulating volume and blood pressure in patients with AKI after major surgery	20 (100)	IV Crystalloid	55	20	22	61	52

*Abbreviations:* *AKI* acute kidney injury, *NSAID* non-steroidal anti-inflammatory drug, *ACEI* angiotensin converting enzyme inhibitor, *ARB* angiotensin receptor blocker.

The results of the pre-meeting survey of local clinicians are also summarized in Table 3. Ninety-five percent of clinicians surveyed indicated that urine output and fluid balance should be monitored daily after surgery. All physicians agreed that intravenous crystalloids should be used to restore circulating blood volume in patients with AKI after major surgery, and 95% agreed that post-surgical patients should not receive diuretics in the absence of volume overload after AKI onset. Ninety-five percent of physicians agreed that patients with AKI should not receive NSAIDs. There was a mixed response to whether patients with AKI should avoid ACE inhibitors or ARBs with 60% agreeing that they should not be prescribed, 15% disagreeing with this statement, and 25% being unsure.

### Barriers and facilitators to AKI prevention, early recognition, and management

The Clinical Practice Guidelines Framework for Improvement [15] was used to categorize major barriers and facilitators to adherence to clinical practice guidelines for AKI raised in discussion with stakeholders. This framework was designed to evaluate knowledge, attitudes, and behavior surrounding barriers and facilitators in knowledge use in health care. The following sections discuss the major barriers and facilitators that were identified by stakeholders. Table 4 provides a complete list of the key barriers and facilitators that were identified during the meeting.

**Table 4:** **Barriers to implementing recommendations for prevention, recognition, and management of Acute Kidney Injury (AKI) following major surgery, according to the clinical practice guidelines framework for improvement**

**Knowledge**	**Attitudes**	**Behaviors**
**Lack of awareness:**	**Interpretation of evidence:**	**Lack of compatibility with current practices:**
● Care providers may not know current definition of AKI based on small change in serum creatinine. Providers may not be aware that a patient has Stage 1 AKI when serum creatinine remains within reference range	● The common research definitions of AKI differ from those often used in clinical care; too many different definitions across research studies	● It is common to give NSAIDs to patients to control pain and increase mobilization, although they may contribute to AKI.
● Providers may not be aware of the mortality and expense associated with AKI; some may not be aware that it is preventative and modifiable	● Uncertainty about whether AKI guideline recommendations apply to post-surgical care	● Many current patients undergoing surgery have comorbidities and use anti-hypertensive medications like ACEI/ARB that may contribute to AKI
● Providers are not always aware of a patient’s risk of AKI after surgery, and if made aware may be more careful to implement monitoring and preventative measures	● Uncertainty about use of medications such as ACE/ARB, diuretics, and NSAIDs and risk of AKI in the perioperative period	● Liver surgery patients are kept “dry” to decrease blood loss and increase transfusion, but the lack of fluids may contribute to AKI.
**Lack of familiarity:**	**Lack of applicability due to patient characteristics:**	● Colorectal surgery patients are also kept “dry” because of a perception that the anastomotic failure rate goes down, but the lack of fluids may contribute to AKI.
● Providers may prescribe ACE inhibitors or ARB following surgery, in particularly restarting these medications when they do not recognize a patient is at risk of AKI	● Belief that AKI guideline recommendations are not generalizable to post-surgical care	Surgeons often withhold IV fluids in the 24–48 hours following surgery because the third space volume will be redistributed and patients retain fluids post-operatively; this practice may contribute to AKI
● It is common to prescribe NSAIDs following surgery to reduce pain; providers prescribing these medications may not be aware of the patient’s risk or history of AKI	● Cardiac surgery patients already received intensive post-operative care in intensive care units, so it less likely that interventions targeting AKI prevention and recognition can improve the quality of care or outcomes in this setting	**Complexity:**
● Providers receive many calls about fluid balance and output, but may lack skills in determining volume status	● Patients undergoing colorectal, hepatic, and some other open abdominal surgeries are often intentionally kept “dry” to aid bowel anastomosis, decrease bleeding, and to avoid volume overload after fluid shifts into third spaces	● AKI is not a discrete entity
● Recognition of AKI will be inadequate if one doesn’t know how to respond	**Concern about cost impacts:**	● Fluid balance (which affects AKI) is extremely complex
**Forgetting:**	● Serum creatinine and physiologically monitoring will increase costs if done repeatedly	● Physicians do not want to put a Foley catheter in every patient that has surgery because there are side effects from this practice, even when it may improve recognition of AKI based on low urine output
● Care providers on surgical services experience many competing demands and are often primarily concerned about surgical performance; providing guidance and recommendations may facilitate better care	● Treating patients that are unlikely to improve can be expensive	● Low urine output has been described in varying ways (eg. with or without standardization by weight and over varying periods of time), but there is uncertainty about the way in which urine output should be measured and recorded, including which is most valid and should be acted upon.
● Forgetting may be particularly common when on-call and during night shifts when tired; residents may be less likely to remember to ask the important questions to guide care decisions when called upon	**Lack of agreement in general:**	**Lack of observability:**
	● Physicians from other specialties may express competing priorities (i.e., cardiologists may not agree with withholding ACEI inhibitors and ARB because they can improve cardiac output post-operatively)	● Physicians do not always see direct links between their practices and prevention/reversal of AKI.
	● While some medication types should generally be withheld, there is a lot of room for judgment and exceptions for a minority of patients	● AKI does not have symptoms like many other diseases to help with identification and prompt treatment.
	**Too “cookbook”/rigid to be applicable:**	**Communication:**
	● Educational initiatives often lack context and are too far removed from the realities of the workplace to be relatable	● There is a lack of mechanisms to facilitate appropriate consultation and communication between care providers
	**Challenge to autonomy:**	● Communication often does not occur promptly and so recommendations are not put in place in a timely manner.
	● There needs to be room for clinical judgment because there are always exceptions	● Nurses routinely notify physicians about low urine output but changes in serum creatinine are rarely communicated
	**Not practical:**	● Nurses may not give feedback to physicians following a therapy or intervention that did not have desired effect.
	● Current guidelines for AKI are not practical because they lack specificity about which patients are “at risk”	**Time pressure:**
	**Lack of expectation involving feelings:**	● Physicians on call receive may calls and thorough evaluation or recognition of AKI risk may not occur prior to ordering medications
	● Some physicians may not perceive AKI as a true threat, but rather, as “just” AKI; interventions may need to take into account these preconceptions	● High volume of patients and busy operating rooms may lower attention to serum creatinine levels
	**Lack of self-efficacy:**	**Lack of Resources:**
	● Some physicians may feel that factors leading to AKI, particularly in elderly patients with complications and/or comorbidities, are not modifiable and therefore, they cannot prevent AKI	● There is a lot of work done by few people; so intensive physiological and laboratory monitoring may not be possible for all patients.
	**Lack of motivation:**	● Physicians must often respond to some problems over the phone rather than at the bedside
	● Physicians may not feel motivated to treat patients that they feel will not improve (due to age or comorbidities)	**Organizational constraints:**
	● Some physicians may lack motivation to prevent AKI because they are not aware of its impact	● In some centers the usual practice may be to consult internal medicine while in others it will be to consult nephrology for AKI, depending on the availability of consult services in the hospital.
		**Lack of access to services:**
		● Some centers may not have electronic health records for reporting and accessing lab results and for computerized clinical decision support for medication prescribing in the setting of reduced kidney function
		● Important information may not be immediately accessible/available when decisions are being made (ie. lab results for the day not yet back, urine output not charted for high risk patients)
		**Shared responsibility with patient:**
		● The risks of developing AKI and its potential consequences may not always been properly conveyed or framed for a patient prior to surgery

*Abbreviations:* *AKI* acute kidney injury, *NSAID* non-steroidal anti-inflammatory drug, *ACEI* angiotensin converting enzyme inhibitor, *ARB* angiotensin receptor blocker.

#### Knowledge

Lack of awareness of risk factors for AKI and criteria for diagnosis of AKI were perceived as important barriers to prevention and early identification. Physicians noted uncertainty about features that could be used to identify patients who were at high risk of AKI, what criteria defined high risk for AKI, and which of several previously published definitions should be used to identify patients with AKI. It was noted that many physicians may not be aware of AKI based on changes in serum creatinine when the serum creatinine remained within the reference range, and so AKI may go unrecognized in the early stages. One participant summarized the problem: “Some of these early changes in serum creatinine may not be apparent if you have a 26 μmol/L or 50% increase in creatinine without triggering an abnormal test result flag on the laboratory report”. Therefore, lack of awareness of patients at high risk and those in the earliest stages of AKI may result in lost opportunities to prevent or reverse AKI soon after its onset.

Lack of familiarity with AKI care was perceived as another barrier to AKI prevention. Participants suggested surgical residents who are less familiar caring for patients with AKI may be more likely to prescribe NSAID for analgesia. One participant stated, “We teach residents…if they have one potentially nephrotoxic drug they shouldn’t get another…but we know it happens”. There was also concern expressed that providers may not include appropriate clinical assessment of volume status before making decisions about administration of intravenous fluids or diuretics. It was felt that education to improve competency of clinical assessment of volume status and identification of medications with effects on kidney function may be helpful.

There was also concern that physicians may forget about the impact of medications on kidney function, which may be especially relevant to busy surgical residents or bedside physicians covering during late night shifts or in the face of competing demands, such as needing to get to the operating room. As one participant noted, “it’s two a.m. in the morning, and you get called about a patient and you forget. You don’t always remember to ask all the questions you should”. Therefore, systems to identify patients at high risk or with AKI, including decision support systems, were identified as potentially valuable.

#### Attitudes

Interpretation of evidence was regarded as a key barrier to knowledge use in clinical care. Referring to the guidelines for AKI identification, one participant stated that, “these guideline definitions have not yet been adopted in clinical settings. They have really been used for the purposes of identifying acute kidney injury in epidemiologic studies”. Participants suggested that the use of different criteria for identification of AKI contributed to variability in care and priorities between the major stakeholder groups. To facilitate interdisciplinary care, they suggested that physicians in critical care, surgery, medicine, and nephrology also needed to establish consensus about how they identified AKI in clinical settings.

Resources and costs were also identified as potential health system barriers. One participant worried that even relatively inexpensive tests like serum creatinine could substantially increase costs if done repeatedly and unnecessarily. Targeting repeated kidney function measurements and more intensive nursing assessment of vital signs, weight, and fluid balance to high risk patients was felt to be an important resource consideration. There was also the concern that providing intensive therapies such as dialysis to patients with AKI who are unlikely to survive may not be appropriate. One participant expressed their concern over the costs of treating these patients by saying, “We owe it to society to be a lot more responsible with the way we use resources because quite frankly, there are often times we are rather wasteful”. The inclusion of a discussion about the risks of AKI and its complications with patients when obtaining informed consent for surgery was proposed as a potential solution.

Clinician autonomy and clinical judgment were highlighted as important facilitators to knowledge use. One participant asserted, “Given the complexity of illness in many patients with AKI, you would not want to take away the ability to use clinical judgment in decision making, but it would be helpful to develop interventions that will support care providers with appropriate knowledge to make the most informed management decisions”. Another participant agreed suggesting that even strict guidance for AKI should allow for clinical judgment: “there will be exceptions to the rule and we should accept it. Fortunately, these are in the minority.” Interventions that support clinical judgment were felt to be an important facilitator for AKI care.

Finally, lack of self-efficacy was identified as a key barrier. It was acknowledged that many physicians may not feel they can prevent AKI, because they do not see a direct link between preventative measures and positive outcomes, and therefore fail to take available precautions. One participant posited, “I think in acute kidney injury there is a bit of nihilism around prevention and management, because there have been many trials of prophylactic and therapeutic agents that have not proven effective”. Another participant agreed: “yes, I think that perception of the condition may be a critical barrier to overcome in order to facilitate uptake of any intervention aimed to prevent or modify the course of AKI”. Acknowledging this, participants agreed that focusing on common, evidence-informed practices for AKI prevention and management, such as appropriate intravenous fluid administration and avoidance or appropriate dose adjustment of drugs with adverse effects of kidney function were identified as priorities to improve safety and quality of care.

#### Behaviors

The lack of compatibility of some AKI prevention and management strategies with common peri-operative practices was identified as a barrier. Examples cited as common practices that address competing priorities of care included: 1) prescribing NSAIDs to post-operative patients to control pain and increase mobilization; 2) using diuretics and inhibitors of the renin-angiotensin system to manage heart failure in cardiac surgery patients, 3) keeping major liver or colorectal surgery patients “dry” during or after surgery to decrease blood loss/minimize blood transfusions and reduce the anastomotic leak rate; and 4) being conservative with intravenous fluids post-operatively following surgery when anticipating redistribution of third space volume back into the intravascular space to reduce volume overload following surgery. These modifiable practices were felt to be important targets for interventions based on intravenous fluid administration and medication use to prevent AKI after major surgery.

Busy clinical schedules and time pressure were also identified as key barriers to improving care for AKI. One participant noted, “I think clinical demands can get overwhelming when you receive so many calls. You may be aware the patient is already on an ACE inhibitor but they require other medicine for their pain and it is easy to start an NSAID”. Other participants agreed that large volumes of high-acuity patients and busy operating rooms may divert attention from patients with rising serum creatinine measures or other early signs of AKI. Lack of time may also contribute to the problem of physicians not being able to provide repeated bedside assessment to assess responses to management implemented in response to recognition of AKI. Ensuring that clinical information is readily available to clinicians was felt to be an important facilitator in the face of competing time demands.

Effective communication between nurses and physicians was identified as a facilitator to AKI prevention and care initiatives on hospital wards. Participants highlighted the importance of information being relayed about changing physiological and laboratory values as well as feedback loops in communication about patients who did not respond to initial management. This was identified as being especially important on surgical wards where physicians round early in the day and may otherwise not be alerted to changes that occur later in the day. Similarly, participants emphasized the importance of communication among physicians, including between the primary physician and consultants as well as at times of transfer of care. One participant commented, “An opinion is of no use if no one rolls up their sleeves and takes action”. Therefore, there may be opportunity for improvement through better communication of the roles and responsibilities of multiple care providers from difference disciplines who are involved in the care of patients with or at risk of AKI after surgery.

### Knowledge translation interventions for AKI prevention, early recognition, and management

The major goals of a knowledge translation (KT) strategy for AKI after major surgery identified by meeting participants were to improve recognition of patients at high risk of AKI, enhance identification of AKI at its earliest stages, encourage use of intravenous fluids and avoidance of medications associated with AKI, and facilitate involvement of specialists in care when indicated. Participants then discussed several strategies that could be used to achieve these goals including formal education, linkage and exchange, audit and feedback, informatics, patient-direct/patient-mediated interventions, organizational, shared decision making, and financial incentives [13]. The majority of the discussion focused on three types of KT interventions for AKI, which participants felt had the greatest potential for further development and evaluation for prevention and early management of AKI after major surgery; 1) formal educational intervention, 2) informatics intervention, and 3) organizational intervention.

#### Formal educational intervention

Gaps in knowledge about AKI were discussed, based on a survey of hospital-based trainees from the UK that reported that 50% of trainees could not define AKI, 30% could not name two or more risk factors for AKI and 37% could not name one or more indication for renal referral [11]. All participants identified education as an important form of intervention. One participant declared, “I believe education should be the basis for any major initiative. Without education, you can’t go anywhere”. On the topic of large group interventions, participants emphasized the importance of starting education about AKI in medical school and sustaining key messages throughout training. Such educational initiatives could be incorporated within surgery and medicine resident academic half days and later through continuing medical education programs. In particular, it was felt that educational initiatives should emphasize modifiable practices for AKI prevention and the outcomes associated with AKI, including mortality and costs.

However, large group educational initiatives were seen as having limited effectiveness when provided in isolation. One participant stated that educational interventions were often “too put together” and too often removed from the realities of clinical practice or omitted practical knowledge and skills to apply in clinical settings. Another participant agreed, saying that information often “just washes right past people”. These concerns highlight the need for educational interventions to tailor important messages and knowledge to the specific audience and their workplace. Small group educational interventions designed to be work-relevant and relatable were recommended. One participant suggested training nurses in small sessions about creatinine changes and when to inform physicians. Residents were a suggested target for interventions because they do much of the ordering of medications and lab testing in teaching hospitals. It was emphasized that residents from all disciplines in medicine and surgery should receive education on AKI prevention and management. It was also suggested that information sessions for residents may best be given by other, more senior residents given that they may be able to provide a more appropriate context for the discussion.

#### Clinical informatics intervention

The role of clinical informatics to identify patients at high risk of AKI and aid in early identification of AKI was discussed. Two recent studies of informatics interventions for AKI were presented. In the first study by McCoy *et al.* [16], a prompt was displayed in an electronic medical record whenever there was a significant change in serum creatinine over the last 48 hours and listed nephrotoxic drugs and medications requiring dose adjustment that a patient was currently prescribed. Physicians were then provided with recommendations to eliminate or adjust the dose of these medications. The study reported that nephrotoxic medications were more likely to be avoided and doses adjusted for kidney function following the introduction of the intervention. The second study by Colpaert *et al.* [17] examined real-time electronic alerts in an intensive care unit, prompted by increases in serum creatinine or decreases in urine output based on the RIFLE criteria. The alert, communicated to physicians via their pagers, was associated with an increase in administration of intravenous fluids. Together, these studies suggested that informatics interventions had the potential to improve processes of care for AKI in hospital settings.

Meeting participants agreed that having an automated alert to identify patients who had developed AKI had high potential as a useful clinical decision support tool. Participants also suggested that tools to flag patients at high risk of AKI before surgery had potential value to make the surgical team aware of this risk and allow providers to tailor the care of these patients. It was suggested that such tools could be based on the type of surgery planned, a prior history of kidney disease or prior episode of AKI, or risk scores for post-operative AKI. Computerized clinical decision support for medication prescribing in the setting of AKI was also identified as a potentially helpful intervention. Although all participants generally expressed interest in the use of such informatics interventions, some participants also cautioned that overuse of electronic pop-ups and alerts in electronic medical records could limit their sustained effectiveness. Most participants agreed that the implementation of such informatics interventions for AKI within clinical workflow was a worthy initiative for further evaluation as prior studies suggested that such strategies may improve identification, appropriateness, and timeliness of interventions for AKI [18].

#### Organizational intervention

Organization interventions incorporating care pathways for early specialist consultation for AKI were also discussed. An observational pilot study by Balasubramanian *et al.* [19] examined the effectiveness of an organizational intervention where patients with early signs of hospital-acquired AKI received early nephrologist care. The study reported that early specialist consultation often resulted in withdrawal or dose adjustment of medications such as ACE inhibitors/ARBs and diuretics, and administration of intravenous fluids. The study reported that early nephrologist consultation was associated with a lower incidence of progression to more severe stages of AKI when compared to a historical control group.

Organizational interventions that included specialist consultation by internal medicine or nephrology were thought to be helpful by most participants. “I could see how a consultant coming when something is triggered would be extremely helpful”, one participant acknowledged. Participants warned that it would be important to establish a clear process for automated consultation and that the admitting surgical team should agree on criteria for consultation and be notified whenever consultations were to be made. Some participants emphasized that consultations performed at the bedside would be preferable to phone-based advice, since they would benefit from a more detailed assessment of the patient including physical examination.

## Conclusions

### Recommendations and implications

At the conclusion of the meeting, three major recommendations were agreed upon by participants. First, participants agreed that patients who had undergone major surgery and were cared for on hospital wards were an appropriate population to target. Specifically, patients undergoing major abdominal and vascular surgery were identified as priority groups because these surgeries carry high risks of AKI, patients who undergo these procedures are not routinely cared for in intensive care units after surgery, and current postoperative care may be more heterogeneous across hospital wards for these patients. Second, participants agreed that KT initiatives for AKI should consider educational interventions, informatics or clinical decision support interventions, and organizational interventions incorporating consultative care for AKI. Finally, the following specific elements were proposed for future implementation and evaluation; 1) identification of patients undergoing elective, urgent, or emergency non-cardiac surgical procedures that carry a high risk of AKI within pre-operative medicine clinics and upon admission to surgical wards, 2) protocols for standardized monitoring of daily serum creatinine, shift urine output, daily fluid balance, and vital sign measurement in high risk patients; 3) automated systems of immediate attending physician alerting upon recognition of the onset of AKI that could be implemented through informatics systems, 4) use of clinical decision support tools for use of IV crystalloids and avoidance of medications commonly used after surgery that worsen kidney function (eg diuretics, NSAIDs, or aminoglycosides), 5) an organizational system to support appropriate timing and indications for consultation with critical care, and nephrology, and 6) an educational program for care providers accompanying the implementation of these KT interventions.

In summary, our stakeholder meeting identified several barriers and potential facilitators to improve uptake of clinical practice guidelines for recognition and management of AKI. Important elements for future KT initiatives were recommended, incorporating education, informatics/clinical decision support, and organizational interventions. Future work will involve designing a KT intervention based on these principles, implementing the intervention, and evaluating its effects on the incidence, processes of care, and outcomes of AKI after major surgery.

## References

[1] De Santo LS, Romano G, Galdieri N, Buonocore M, Bancone C, De Simone V, Della Corte A, Nappi G (2010). RIFLE criteria for acute kidney injury in valvular surgery. J Heart Valve Dis.

[2] Borthwick E, Ferguson A (2010). Perioperative acute kidney injury: risk factors, recognition, management, and outcomes. BMJ.

[3] Hobson CE, Yavas S, Segal MS, Schold JD, Tribble CG, Layon AJ, Bihorac A (2009). Acute kidney injury is associated with increased long-term mortality after cardiothoracic surgery. Circulation.

[4] Hsu C-Y, McCulloch CE, Fan D, Ordoñez JD, Chertow GM, Go AS (2007). Community-based incidence of acute renal failure. Kidney Int.

[5] Xue JL, Daniels F, Star RA, Kimmel PL, Eggers PW, Molitoris BA, Himmelfarb J, Collins AJ (2006). Incidence and mortality of acute renal failure in Medicare beneficiaries, 1992 to 2001. J Am Soc Nephrol.

[6] Siddiqui NF, Coca SG, Devereaux PJ, Jain AK, Li L, Luo J, Parikh CR, Paterson M, Philbrook HT, Wald R, Walsh M, Whitlock R, Garg AX (2012). Secular trends in acute dialysis after elective major surgery – 1995 to 2009. Can Med Assoc J.

[7] Mehta RL, Pascual MT, Soroko S, Chertow GM, Group PS (2002). Diuretics, mortality, and nonrecovery of renal function in acute renal failure. JAMA.

[8] Ftouh S, Thomas M (2013). Acute kidney injury : summary of NICE guidance. BMJ.

[9] Kellum AJ (2012). Offical Journal of the International Society of Nephrology KDIGO Clinical Practice Guideline for Acute Kidney Injury. Kidney Int.

[10] Stewart J, Findlay G, Smith N, Kelly K, Mason M (2009). Adding Insult to Injury Adding Insult to Injury: a review of the care of patients who died in hospital with a primary diagnosis of acute kidney injury (acute renal failure).

[11] Muniraju TM, Lillicrap MH, Horrocks JL, Fisher JM, Clark RMW, Kanagasundaram NS (2012). Diagnosis and management of acute kidney injury: deficiencies in the knowledge base of non-specialist, trainee medical staff. Clin Med.

[12] James M, Bouchard J, Ho J, Klarenbach S, LaFrance JP, Rigatto C, Wald R, Zappitelli M, Pannu N (2013). Canadian Society of Nephrology commentary on the 2012 KDIGO clinical practice guideline for acute kidney injury. Am J Kidney Dis.

[13] Straus SE, Tetroe J, Graham ID (2009). Knowledge Translation in Health Care: Moving from Evidence to Practice.

[14] Walter I, Nutley S, Davies H (2003). Developing a Taxonomy of Interventions used to Increase the Impact of Research.

[15] Cabana MD, Rand CS, Powe NR, Wu AW, Wilson MH, Abboud PA, Rubin HR (1999). Why don’t physicians follow clinical practice guidelines? A framework for improvement. JAMA.

[16] McCoy AB, Waitman LR, Gadd CS, Danciu I, Smith JP, Lewis JP, Schildcrout JS, Peterson JF (2010). A computerized provider order entry intervention for medication safety during acute kidney injury: a quality improvement report. Am J Kidney Dis.

[17] Hoste EA, Benoit D, De Turck F, Decruyenaere J (2012). Impact of real-time electronic alerting of acute kidney injury on therapeutic intervention and progression of RIFLE class*. Crit Care Med.

[18] Kawamoto K, Houlihan CA, Balas EA, Lobach DF (2005). Improving clinical practice using clinical decision support systems: a systematic review of trials to identify features critical to success. BMJ.

[19] Balasubramanian G, Al-Aly Z, Moiz A, Rauchman M, Zhang Z, Gopalakrishnan R, Balasubramanian S, El-Achkar TM (2011). Early nephrologist involvement in hospital-acquired acute kidney injury: a pilot study. Am J Kidney Dis.

